# ACE2 and COVID-19 Susceptibility and Severity

**DOI:** 10.14336/AD.2021.0805

**Published:** 2022-04-01

**Authors:** Ming Zheng

**Affiliations:** ^1^Institute of Military Cognition and Brain Sciences, Academy of Military Medical Sciences, Beijing, China; ^2^Beijing Institute of Basic Medical Sciences, Beijing, China

**Keywords:** COVID-19, SARS-CoV-2, ACE2, host cell receptor, viral tropism, GWAS, cis-eQTL, scRNA-seq, Mendelian randomization

## Abstract

Sick, male, and older populations are more vulnerable to COVID-19. However, it remains unclear whether a common mechanism exists across different demographic characteristics. SARS-CoV-2 infection is initiated by the specific binding of the viral spike protein to angiotensin-converting enzyme 2 (ACE2). This study analyzed the demographics of pulmonary ACE2 expression, Mendelian randomization (MR) of ACE2 and COVID-19, and comparative tropism of SARS-CoV-2. The key features of SARS-CoV-2 tropism, including pulmonary ACE2 expression and ACE2-expressing cell types, showed distinct subphenotypes associated with the demographics of vulnerable COVID-19 populations, suggesting a hypothesis centered on “ACE2” to explain their interplay. Next, by integrating multiple COVID-19 cohorts of genome-wide association studies (GWASs) and cis-expression quantitative trait loci (cis-eQTLs) of ACE2, MR analysis demonstrated that ACE2 played a causal role in COVID-19 susceptibility and severity, suggesting ACE2 as a promising target for early COVID-19 treatment. Next, by analyzing the expression of host cell receptors using single-cell RNA sequencing (scRNA-seq) data of human lung tissues, comparative tropism analysis showed that SARS-CoV-2 and other respiratory viruses, but not non-respiratory viruses, had remarkably overlapping and enriched cellular tropism in alveolar type 2 (AT2) cells. This finding indicates the possibility of coinfection with SARS-CoV-2 and other respiratory viruses, perhaps implying sociovirology at the cellular level. Moreover, the binding of viral entry proteins to the compatible host cell receptors is under strong natural selection pressure. Therefore, comparative tropism might reveal the footprint of natural selection that shapes the virus population, which provides a novel perspective for understanding zoonotic spillover events.

Since 2020, the coronavirus disease 2019 (COVID-19) outbreak has become a global pandemic. To date, the number of COVID-19 worldwide has exceeded 200 million (https://covid19.who.int/). It has been reported that COVID-19 patients with demographic characteristics of pre-existing disease, male sex, and older age are particularly prone to severe clinical outcomes, such as hospitalization, ICU admission, and death [1]. In the U.S., patients who die from COVID-19 are predominantly individuals with pre-existing diseases (96.4%) and aged more than 50 years (95.4%) [1]. Despite accumulating evidence demonstrating the substantial variation in the COVID-19 outcomes across demographics, it remains unclear whether a common mechanism exists across different demographic characteristics, including medical conditions, sex, and age.

COVID-19 is caused by a novel coronavirus, designated severe acute respiratory syndrome coronavirus 2 (SARS-CoV-2) [2]. SARS-CoV-2 infection is initiated by the specific binding of the viral spike protein to angiotensin-converting enzyme 2 (ACE2) [3]. It is worth noting that the expression of ACE2 is associated with increased viral load in human cell lines [4, 5] and mice [6]. Moreover, ACE2 expression has been linked to the clinical consequences of COVID-19 [7]. Thus, ACE2 expression plays an essential role in both SARS-CoV-2 infection and COVID-19 manifestations. However, the fundamental question still exists whether ACE2 expression varies across demographics, and the role of ACE2 in COVID-19 remains unclear.

**Figure 1.**
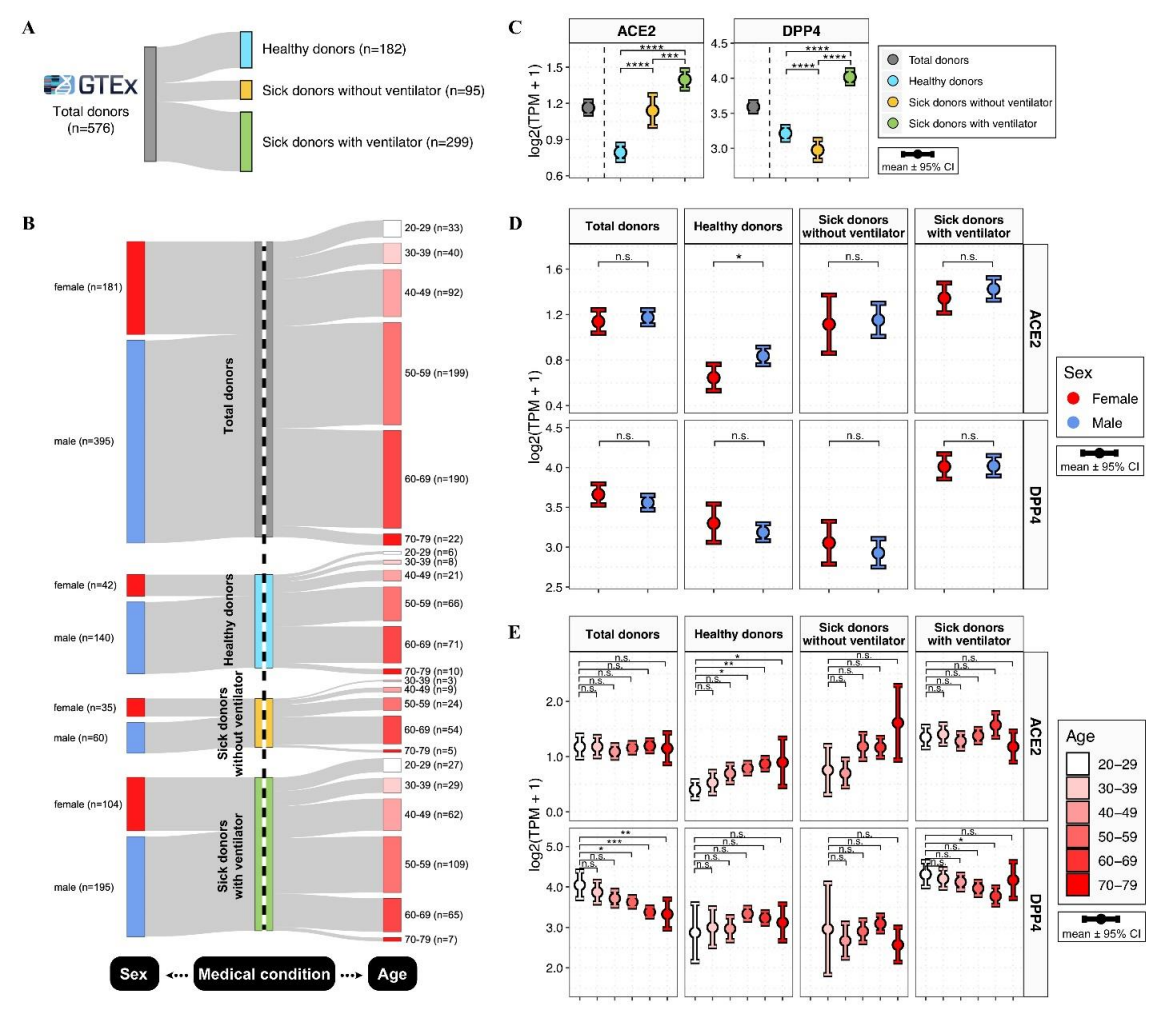
Demographics of pulmonary ACE2 and DPP4 expression. (A-B) Sankey plots show the sample sizes of GTEx donors across demographics, including medical conditions, sex, and age. (C-E) Distributions of ACE2 and DPP4 expression levels in lung tissues among all donors and the subgroups defined by medical condition (C), sex (D), and age (E). Expression levels were measured by log2-transformed TPM (transcripts per million) values. The error bars represent the 95% confidence interval (CI), with color coding according to medical status (C), sex (D), and age (E). The results were considered statistically significant when P < 0.05 (*), P < 0.01 (**), P < 0.001 (***), and P < 0.0001 (****) using the Kruskal-Wallis test.

## Pulmonary ACE2 expression across demographics

ACE2 is the host cell receptor for both SARS-CoV [8] and SARS-CoV-2 [3]. Both of these viruses cause viral pneumonia, which is the primary reason for fatality [9, 10]. Previously, positive ACE2 expression in human lung tissues was detected using immunohistochemistry staining [11]. To extend the previous finding, we investigated the expression pattern of ACE2 in human lung tissues across demographic factors of medical condition, sex, and age.

Here, we analyzed the RNA-seq data from the GTEx cohort, which contains individuals of predominantly European ancestry [12]. ACE2 expression was analyzed in 576 human lung tissue samples from 182 healthy donors and 394 sick donors naive to SARS-CoV-2 infection (Fig. 1A). Sick donors suffered from chronic illnesses, including cerebrovascular, cardiac, hepatic, renal, neurological and respiratory diseases. These common diseases were largely consistent with the pre-existing diseases reported in COVID-19 patients [1]. Additionally, the 394 sick donors included 95 sick donors without ventilator usage and 299 sick donors with the therapeutic intervention of ventilator usage (Fig. 1A-B). Compared to the healthy donors, the sick donors, with or without ventilator support, had significantly higher ACE2 expression levels (Fig. 1C, left plot). Moreover, sick donors with ventilator support had markedly higher ACE2 expression levels than sick donors without ventilator support.

**Figure 2.**
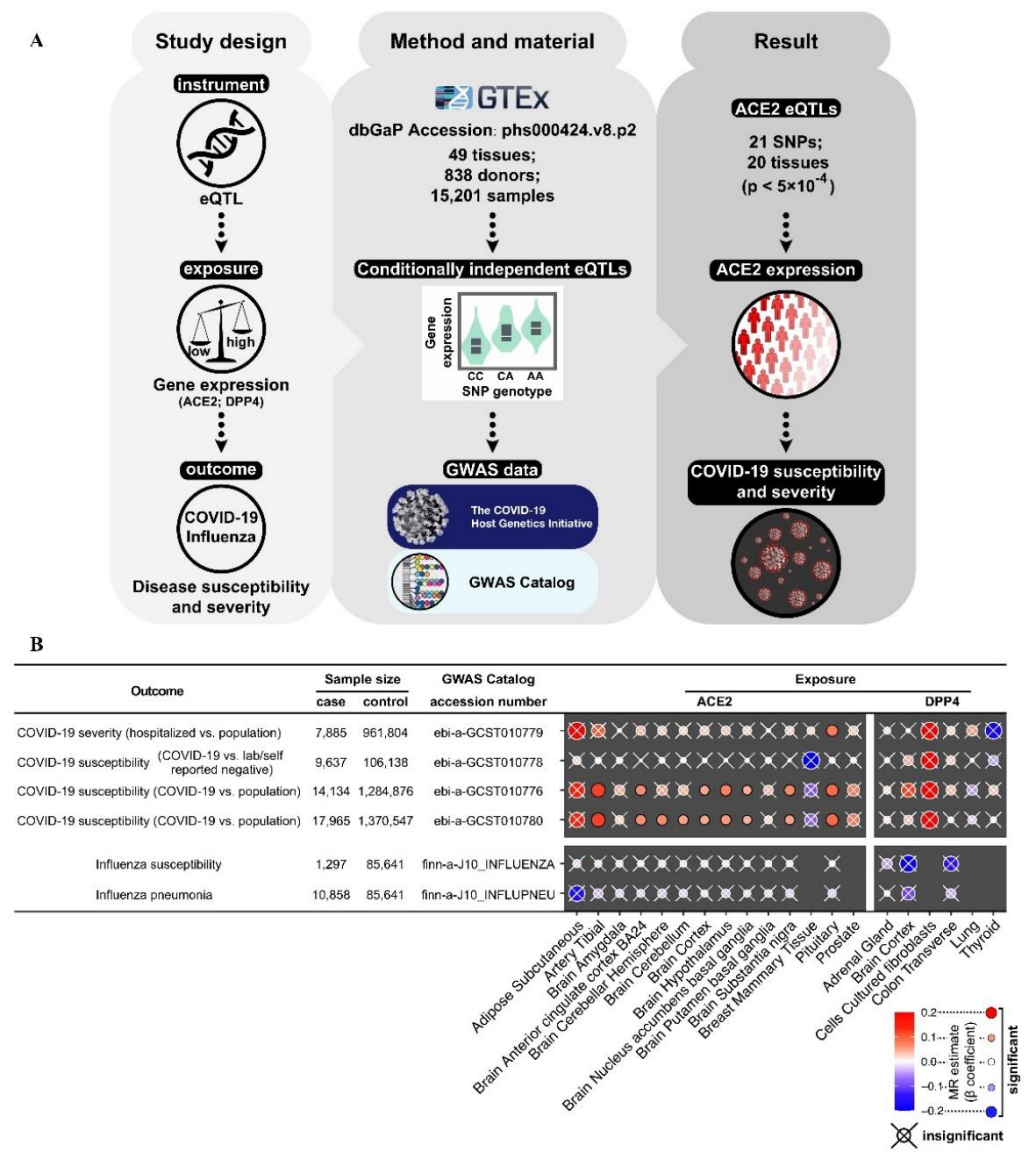
Mendelian randomization study of ACE2 and COVID-19 susceptibility and severity. (A) Graphical representation of the study design, method and material, and result of the Mendelian randomization (MR) study. This MR study used expression quantitative trait loci (eQTLs) to estimate the causal role of ACE2 expression in COVID-19 susceptibility and severity. This MR analysis tested the cis-eQTL proposed instruments of ACE2 and DPP4 against multiple genome-wide association study (GWAS) data of COVID-19 and influenza. (B) MR results show the causal role of ACE2 in the susceptibility and severity of COVID-19 but not influenza. MR estimates were calculated using inverse-variance weighting (IVW) for instruments that contained more than one cis-eQTL and the Wald ratio for instruments with one cis-eQTL. Thus, instruments containing only one cis-eQTL were not tested for IVW. Heatmap shows MR estimates available in at least one tissue and GWAS cohort. The color and size of dots represent the β estimate of the MR. A positive β estimate indicates that high gene expression is associated with an increased risk of disease susceptibility and severity. The results were considered statistically significant when P?<?0.05, and the “×” represents insignificant results.

**Figure 3.**
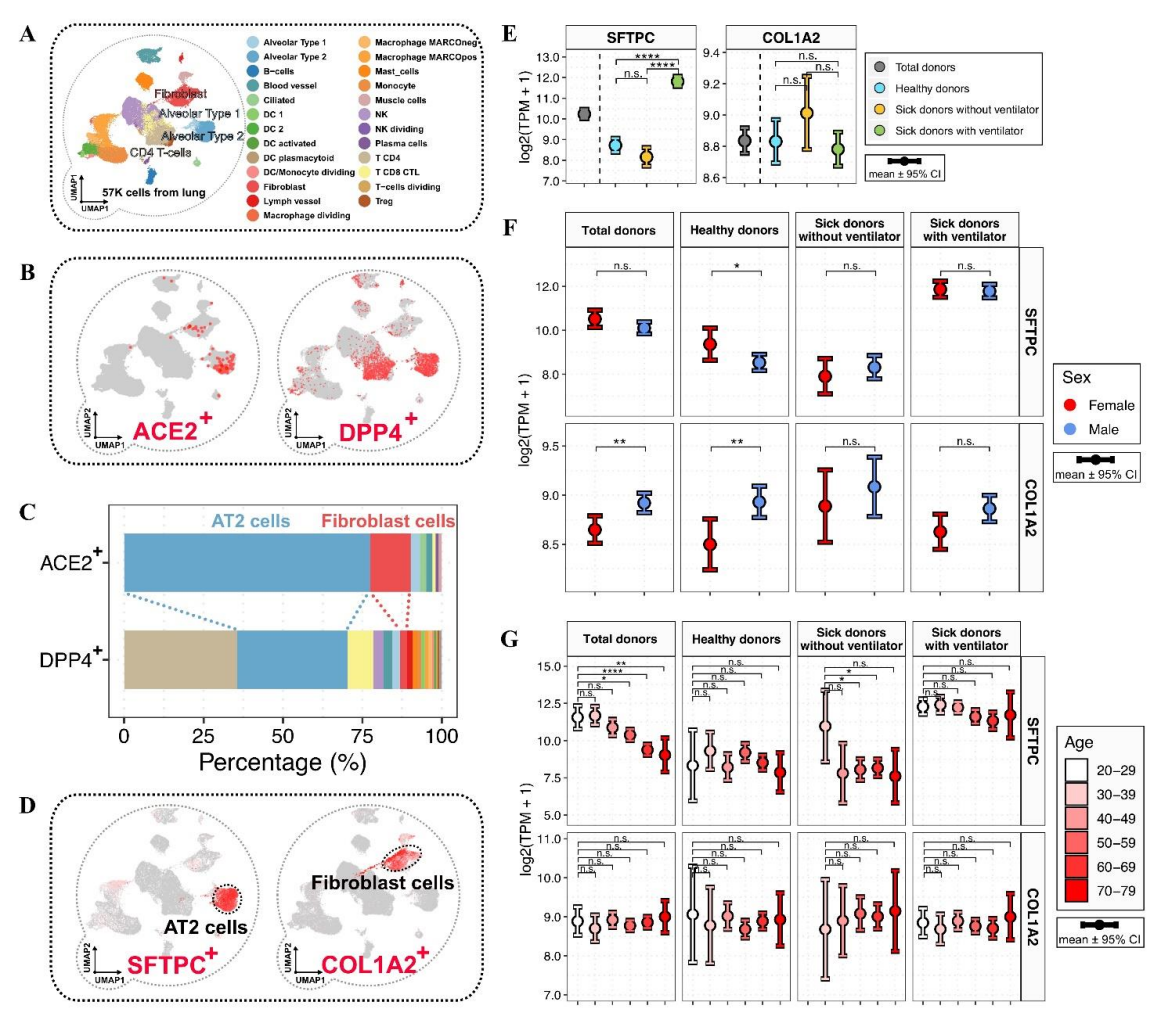
Pulmonary ACE2^+^ and DPP4^+^ cell types and the relative abundances of alveolar type 2 and fibroblast cells across demographics. (A) The uniform manifold approximation and projection (UMAP) plot shows the landscape of lung cells at the single-cell level, with color coding according to cell type. (B) UMAP plots show the expression levels of ACE2 and DPP4, with color coding according to the gene expression level. (C) The stack bar plot shows the percentages of pulmonary ACE2^+^ and DPP4^+^ cells, with color coding according to cell type. (D) UMAP plots show the expression levels of SFTPC and COL1A2, which were exclusively expressed in alveolar type 2 (AT2) and fibroblast cells, respectively. (E-G) Distributions of pulmonary SFTPC and COL1A2 expression levels in total donors and the subgroups defined by medical condition (E), sex (F), and age (G). The error bars represent the 95% confidence interval (CI), with color coding according to medical status (E), sex (F), and age (G). The results were considered statistically significant when P < 0.05 (*), P < 0.01 (**), P < 0.001 (***), and P < 0.0001 (****) using the Kruskal-Wallis test.

Next, we explored the sex- and age-related differences in ACE2 expression. Among all donors, males had slightly higher ACE2 expression levels than females, but the difference was insignificant (Fig. 1D, upper left plot). Next, among all subgroups defined by medical condition, ACE2 expression levels were upregulated in males compared with females, but a statistically significant difference was observed only in the healthy subgroup (Fig. 1D, upper plot). Additionally, the ACE2 expression levels were significantly increased with age only in the healthy subgroup (Fig. 1E, upper plot).

In parallel, we analyzed the expression of dipeptidyl peptidase 4 (DPP4), the host cell receptor for Middle East respiratory syndrome coronavirus (MERS-CoV) [13]. Compared to the healthy donors, the DPP4 expression levels were significantly lower in the sick donors without ventilator support but remarkably higher in the sick donors with ventilator support (Fig. 1C, right plot). Moreover, DPP4 expression showed no significant sex-related difference (Fig. 1D, lower plot). The DPP4 expression levels were significantly decreased with age among all donors and the subgroup of sick donors with ventilator support (Fig. 1E, lower plot). Thus, ACE2 and DPP4 had different expression patterns across demographics, indicating different tissue tropisms between SARS-CoV-2 and MERS-CoV.

The above results revealed upregulated ACE2 expression levels in sick, male, and older populations before SARS-CoV-2 infection. It has been reported that the demographic charateristics of pre-existing diseases, male sex, and older age are risk factors for severe COVID-19 [1]. Thus, the expression of ACE2 coincides with the clinical manifestations of COVID-19 across demographics, suggesting a hypothesis centered on “ACE2” for COVID-19 risk.

## Mendelian randomization study of ACE2 and COVID-19 susceptibility and severity

To better understand the relationship between ACE2 and COVID-19 risk, we conducted a Mendelian randomization (MR) study. MR, built on Mendel’s second law, is a commonly used genetics technique that implicates the causality of exposure for a disease outcome [14]. MR analysis uses genetic variants as instrumental variables. Since genetic variants are randomly assorted during meiosis and typically unassociated with potential confounders, MR results are less prone to the bias of confounding and reverse causality [14].

Here, we conducted MR to infer the causal effect of ACE2 on COVID-19 manifestations, including disease susceptibility and severity. Fig. 2A describes the study design, method and material, and the results of our MR study. Two-sample MR analysis was conducted using TwoSampleMR software (https://mrcieu.github.io/TwoSampleMR/), as previously reported [15]. We selected conditionally independent cis-expression quantitative trait loci (cis-eQTLs; *p*-value < 5×10^-4^) of ACE2 and DPP4 from the GTEx v8 dataset [16]. Linkage disequilibrium (LD) clumping was performed for pruning correlated cis-eQTLs (r^2^ < 0.001) using the PLINK clumping method [17, 18]. Next, we tested the cis-eQTL proposed instruments of ACE2 and DPP4 against multiple genome-wide association study (GWAS) data of COVID-19 and influenza from the COVID-19 Host Genetics Initiative [19] and GWAS Catalog [20]. The GWAS cohorts were categorized into disease susceptibility and severity (Fig. 2B). COVID-19 severity was indicated by hospitalization, while influenza severity was indicated by influenza pneumonia.

MR estimates were calculated using inverse-variance weighting for instruments that contained more than one cis-eQTL and Wald ratio for instruments with one cis-eQTL. All the significant MR estimates (β-coefficients) of ACE2 were positive for both the susceptibility and severity of COVID-19 (Fig. 2B), indicating that upregulated ACE2 expression is associated with an increased risk of COVID-19. In contrast, all the MR β-coefficients for DPP4 exposure and influenza outcome were insignificant. Thus, our MR study discovered that ACE2 played a causal role in COVID-19 susceptibility and severity, indicating ACE2 as a potential therapeutic target of COVID-19.

## Overlapping virus-permissive cells between MERS-CoV and SARS-CoV-2

The cellular expression of host cell receptors is a prerequisite of virus infection, and thus forms the basis of the understanding of on which cells a virus can exert its pathogenic effects. Based on this theory, previous studies have demonstrated the feasibility of mapping virus-permissive cells using single-cell RNA-seq data (scRNA-seq) [7, 21, 22]. Here, by analyzing the scRNA-seq data of 57,020 cells from healthy human lung tissues from a previous study [23], we investigated the identities of ACE2^+^ and DPP4^+^ cells (Fig. 3A-B). According to the original study, we retained the information of cell clustering and cell type annotations (Fig. 3A) [23]. Compared to ACE2, DPP4 was expressed by a broader distribution of cell types (Fig. 3B-C). The most dominant cell types for ACE2^+^ cells were alveolar type 2 (AT2) and fibroblast cells (Fig. 3C). It is worth noting that 77.5% of ACE2^+^ cells and 34.8% of DPP4^+^ cells were AT2 cells, albeit at much lower proportions, 12.7% of ACE2^+^ cells and 2.2% of DPP4^+^ cells were fibroblast cells (Fig. 3C). These results indicate the substantial overlap of virus-permissive cells between MERS-CoV and SARS-CoV-2.

## Relative abundances of pulmonary AT2 and fibroblast cells across demographics

Due to the dominant proportion of AT2 and fibroblast cells in ACE2^+^ cells, we investigated whether the relative abundances of AT2 and fibroblast cells were related to medical condition, sex, and age. In the scRNA-seq data of lung tissues, surfactant protein C (SFTPC) and collagen type I alpha 2 chain (COL1A2), the well-acknowledged cell markers of AT2 and fibroblast cells, were expressed dominantly by AT2 and fibroblast cells (Fig. 3D). Next, we measured the relative abundances of AT2 and fibroblast cells through the expression of SFTPC and COL1A2 in lung tissues. We observed quantitative fluctuations in AT2 and fibroblast cell abundances in sick, male, and older populations (Fig. 3E-G), indicating the importance of investigating both ACE2 expression and ACE2-expressing cell types.

**Figure 4.**
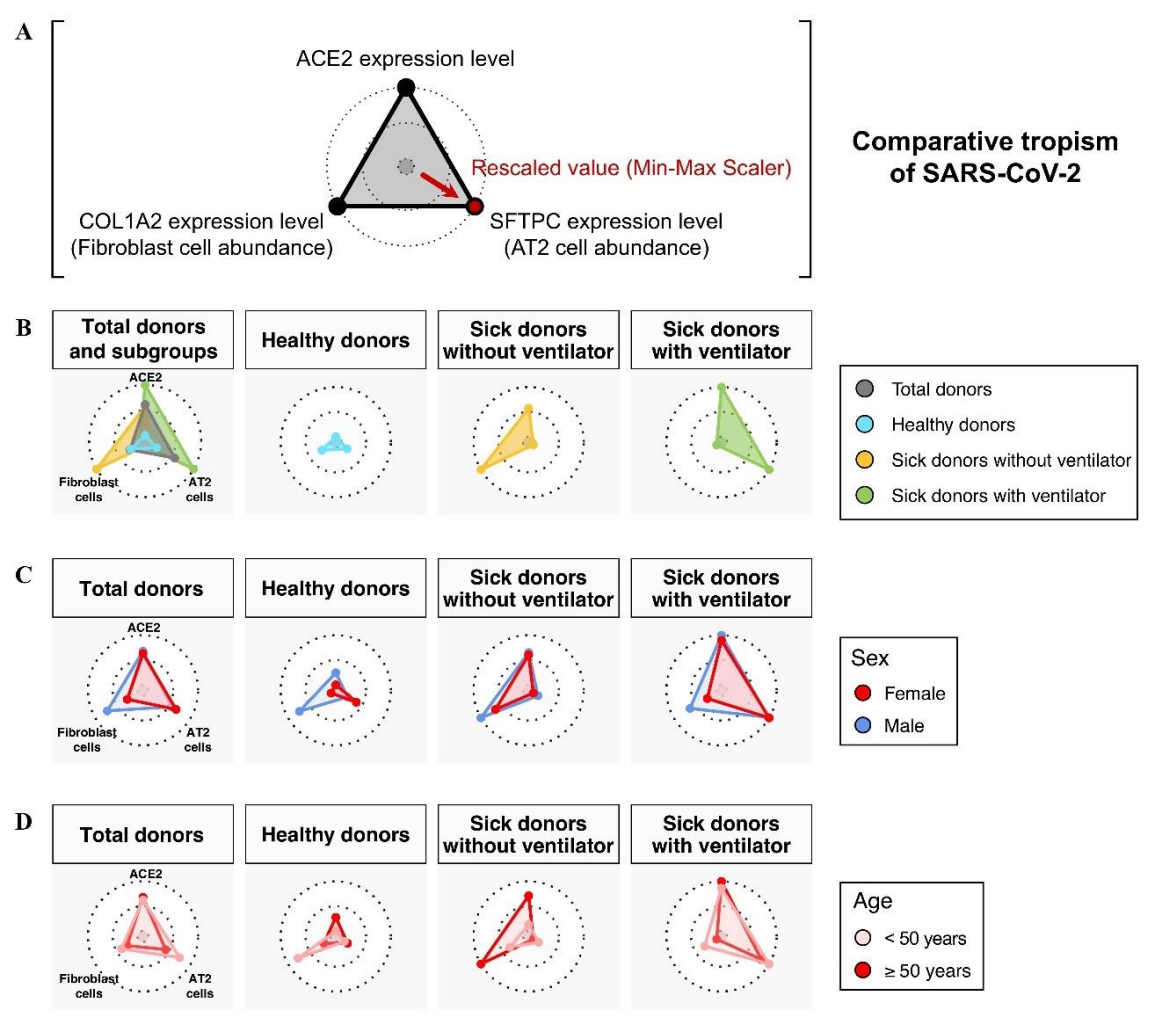
Key features of viral tropism for SARS-CoV-2 across demographics. (A) Comparative analysis of viral tropism for SARS-CoV-2 through phenotyping the ACE2 expression level and relative abundances of AT2 and fibroblast cells. The relative abundances of AT2 and fibroblast cells were measured by the expression of their cell markers, SFTPC and COL1A2. The average expression levels of ACE2, SFTPC, and COL1A2 were scaled using the min-max scaler (MMS) method for radar charts. (B-D) Radar charts show the feature variables of SARS-CoV-2 tropism (ACE2 expression level, relative abundances of AT2 and fibroblast cells) in total donors and the subgroups defined by medical condition (B), sex (C), and age (D).

To capture the above-stated changes in the key features of viral tropism for SARS-CoV-2, we used radar charts to graphically represent the feature variables of SARS-CoV-2 tropism, including ACE2 expression and the most dominant SARS-CoV-2-permissive cells, AT2 and fibroblast cells (Fig. 4A). The radar charts successfully capture the distinct patterns of SARS-CoV-2 tropism across the sick, male, and older populations (Fig. 4B-D). Compared to the healthy subgroup, the ACE2 expression levels were increased in the sick subgroups and increased even more in the sick subgroup with ventilator usage (Fig. 4B). Of note, ventilator usage was associated with an inverse relationship in the abundances of AT2 and fibroblast cells. Next, we found that males had increased fibroblast cell abundances and higher ACE2 expression levels than females. These findings were concordant among all donors as well as all three subgroups defined by medical condition (Fig. 4C). In contrast, we observed no concordant age-related differences in the ACE2-expressing cells, whereas consistently higher ACE2 expression levels were observed in the older subgroup (≥ 50 years) than in the younger subgroup (< 50 years) (Fig. 4D).

## Overlapping cellular tropism in human respiratory viruses

**Figure 5.**
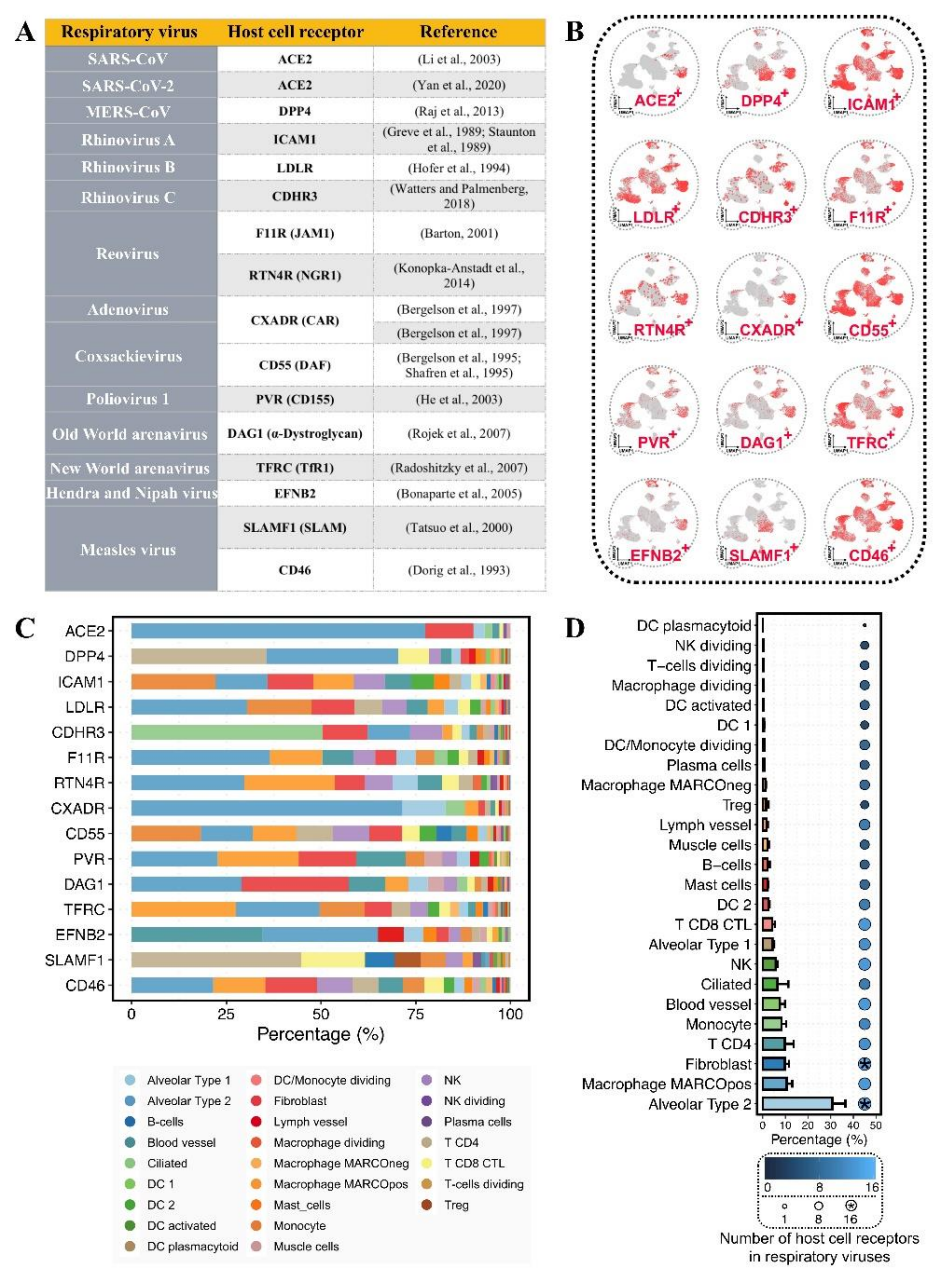
Overlapping cellular tropism in human respiratory viruses. (A) Respiratory viruses and the host cell receptors. (B) UMAP plots show the pulmonary expression of host cell receptors from human respiratory viruses. (C) The percentages of virus-permissive cell types with the expression of host cell receptors. (D) Bar plot shows the average percentages of virus-permissive cell types for respiratory viruses. The error bar indicates the standard error. The dot color and size represent the number of viral-specific host cell receptors expressed by the cell type. The “*” shape represents the cell type expressing all the host cell receptors.

The global view of a comparative tropism across human viruses is still lacking. This hole in our knowledge is due in part to the technical difficulty of investigating comparative tropism systematically. Recently, we developed a bioinformatic framework for mapping viral tropism through the expression of host cell receptors [7]. Here, by applying our bioinformatic framework to the scRNA-seq data of human lung tissues from the previous study [23], we conducted a comparative tropism analysis across different human respiratory viruses. We characterized the virus-permissive cells in 14 human respiratory viruses, including SARS-CoV, SARS-CoV-2, MERS-CoV, rhinovirus A, rhinovirus B, rhinovirus C, reovirus, adenovirus, coxsackievirus, poliovirus 1, Old World arenavirus, New World arenavirus, Hendra and Nipah viruses, and measles virus (Fig. 5A). These respiratory viruses employ various host cell receptors, including ACE2 [3, 8], DPP4 [13], ICAM1 [24, 25], LDLR [26], CDHR3 [27], F11R [28], RTN4R [29], CXADR [30], CD55 [31, 32], PVR [33], DAG1 [34], TFRC [35], EFNB2 [36], SLAMF1 [37], and CD46 [38]. Using scRNA-seq data from healthy human lung tissues, we analyzed the percentage of virus-permissive cells with the expression of different host cell receptors (Fig. 5B-C). Notably, all these respiratory viruses showed overlapping cellular tropisms in AT2 and fibroblast cells. Moreover, AT2 and fibroblast cells were the first and third most dominant cell types, respectively (Fig. 5D).

## Comparative cellular tropism between human respiratory and non-respiratory viruses

**Figure 6.**
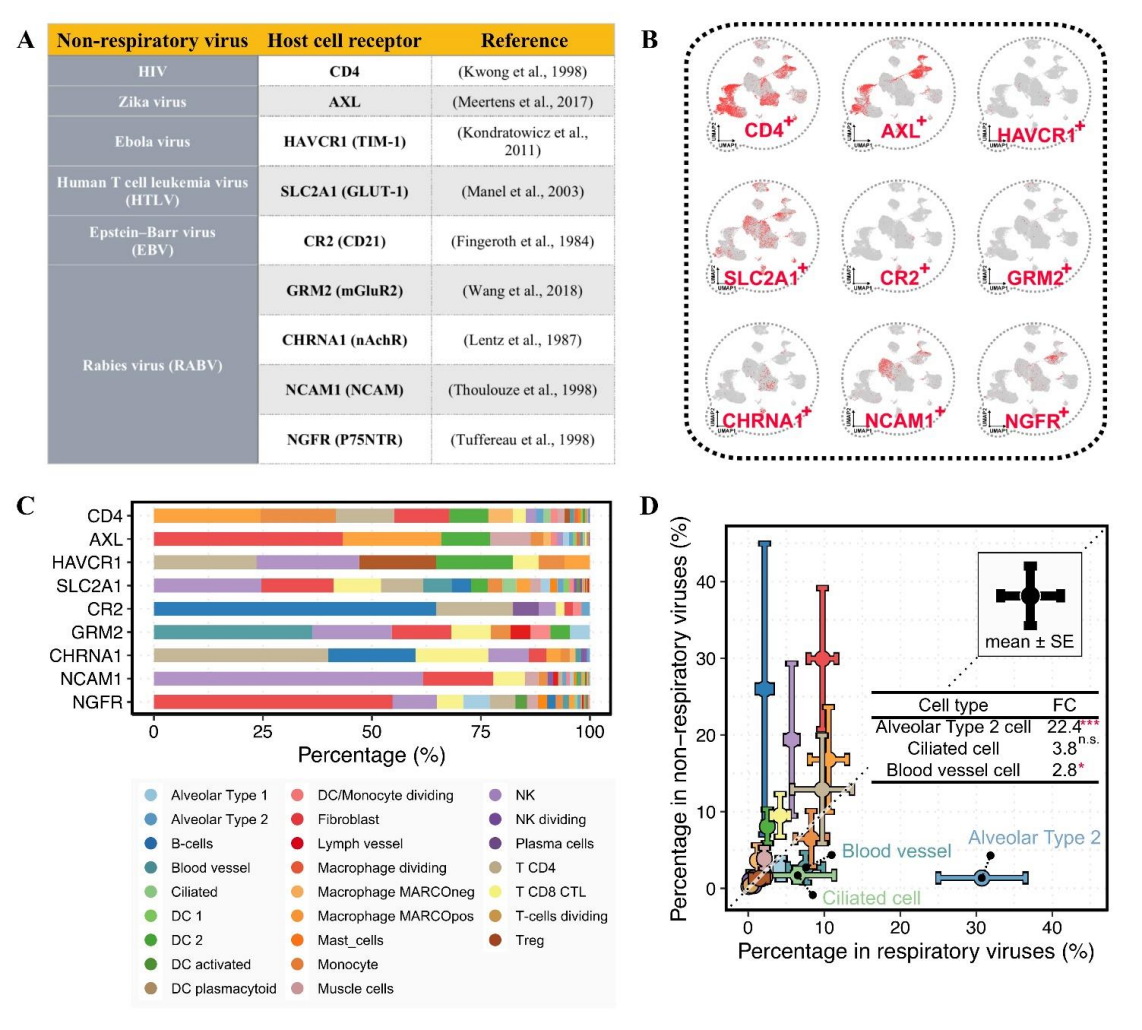
Comparative cellular tropism between respiratory and non-respiratory viruses. (A) Non-respiratory viruses and the host cell receptors. (B) UMAP plots show the pulmonary expression of host cell receptors from human non-respiratory viruses. (C) The percentages of virus-permissive cell types with the expression of host cell receptors. (D) Comparison between the average percentages of virus-permissive cell types for respiratory and non-respiratory viruses. The error bar indicates the standard error. The dashed line represents the 1.0 slope of complete concordance. The result was considered statistically significant when P < 0.05 (*), P < 0.01 (**), P < 0.001 (***), and P < 0.0001 (****) using the Kruskal-Wallis test. FC represents fold change.

Next, we investigated the comparative cellular tropism between human respiratory and non-respiratory viruses. We analyzed six non-respiratory viruses for comparison, including human immunodeficiency virus (HIV), Zika virus, Ebola virus, human T cell leukemia virus (HTLV), Epstein-Barr virus (EBV), and rabies virus (RABV) (Fig. 6A). The corresponding host cell receptors included CD4 [39], AXL [40], HAVCR1 [41], SLC2A1 [42], CR2 [43], GRM2 [44], CHRNA1 [45], NCAM1 [46], and NGFR [47]. By analyzing the expression of host cell receptors, we calculated the percentage of virus-permissive cells for human non-respiratory viruses (Fig. 6B-C). The average proportion of AT2 cells was 22.4-fold higher in respiratory viruses than in non-respiratory viruses (*p*-value < 0.001, Fig. 6D), but no significant difference was observed in fibroblast cells. This finding implies the important role of virus-permissive AT2 cells in respiratory rather than non-respiratory viruses.

## Relationships between heterogeneous ACE2 expression, COVID-19 manifestations, and comparative tropism of SARS-CoV-2 across demographics

**Figure 7.**
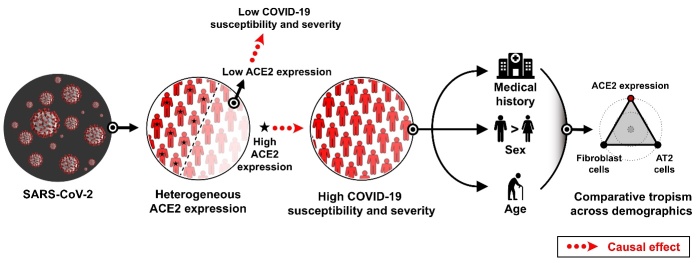
Causal role of ACE2 in COVID-19 susceptibility and severity, and comparative tropism of SARS-CoV-2 across demographics. Sick, male, and older populations have higher ACE2 expression levels, leading to increased COVID-19 susceptibility and severity. The current definitions of severe COVID-19 populations, including sick, male, and older populations, might reflect the subphenotypes of SARS-CoV-2 tropism across demographics. The dashed arrow shows the causal effect of ACE2 on COVID-19 susceptibility and severity.

Due to the causal role of ACE2 in COVID-19, sick, male, and older populations with higher ACE2 expression levels were more vulnerable to SARS-CoV-2 infection, leading to higher COVID-19 susceptibility and severity among these subgroups (Fig. 7). Therefore, ACE2 is centrally associated with the demographic risk factors for COVID-19. Next, comparative tropism analysis revealed the demographic features of susceptible cell types for SARS-CoV-2 infection, indicating fundamental changes in the virus-host interaction across demographics.

A previous study reported the causal role of ACE2 in COVID-19 severity through MR analysis [15]. In this study, we discovered that ACE2 had a significant causal role in not only the severity but also the susceptibility to COVID-19. It is worth noting that, although both previous and our MR studies show strong evidence that higher ACE2 expression increases the risk of COVID-19, both studies have a limitation in that no pulmonary cis-eQTL instrument was available for analyzing ACE2 in lung tissues. Nevertheless, both previous and our MR results strongly suggest ACE2 as one of the most promising actionable and druggable targets for the early treatment of COVID-19.

ACE2 is the prerequisite of viral infection for both SARS-CoV [8] and SARS-CoV-2 [3]. This study found that a substantial proportion (77.5%) of pulmonary ACE2^+^ cells were AT2 cells. AT2 cells, rather than AT1 cells, are preferentially infected by both SARS-CoV and SARS-CoV-2 [5, 48]. Moreover, in a postmortem pathological study of a ready-for-discharge COVID-19 patient, SARS-CoV-2 virus nucleic acid was negative in nasopharyngeal swabs but positive in the lungs, and electron microscopic testing clearly showed coronavirus particles in AT2 cells, implying that AT2 cells retain SARS-CoV-2 infection for prolonged periods [49]. Thus, ACE2-expressing cells successfully indicate the infectable cell types, and the proportional distribution of those virus-permissive cells has profound implications for the preference of viral infection.

In this study, we discovered that donors on mechanical ventilation had the most remarkably increased ACE2 expression levels and AT2 cell abundances. It has been reported that mechanical ventilation causes the proliferation of AT2 cells [50], which might partly explain the increased abundance of AT2 cells. Recently, there has been a strong argument regarding whether ventilator support should be employed early in COVID-19 patients to prevent disease progression [51]. The major concern for this argument is that vigorous mechanical ventilation can rapidly lead to ventilator-induced lung injury. It is worth noting that AT2 cells play a critical role in decreasing lung injury through controlling the immune response and repairing the injured lung [52]. However, AT2 cells are the primary target of SARS-CoV-2 infection. COVID-19 patients with SARS-CoV-2-infected and impaired AT2 cells might be particularly vulnerable to ventilator-induced lung injury. Therefore, ventilator support in patients with early COVID-19 should be used cautiously, and further clinical study on this issue is required.

It has been reported that the influenza virus preferentially infects AT2 cells [53], and AT2 cells maintain the replication of influenza virus for more prolonged periods [54]. This evidence indicates the possibility of coinfection between SARS-CoV-2 and influenza virus at the cellular level. A previous study found that the coinfection rate of SARS-CoV-2 and influenza virus was as high as 57.3% during the COVID-19 outbreak period in Wuhan [55]. Moreover, coinfection with influenza virus significantly enhances SARS-CoV-2 infection, leading to more severe lung damage in mice [56]. Thus, when the current COVID-19 pandemic merges with the flu season, the coinfection of influenza virus and SARS-CoV-2 might cause a more severe threat to public health.

Furthermore, AT2 cells are also the preferential host cells for other respiratory viruses. Through the comparative tropism analysis of 14 respiratory viruses, we found that, first, albeit using different host cell receptors, all the respiratory viruses showed overlapping cellular tropism to the AT2 cells; second, among the heterogeneous host cells targeted by respiratory viruses, the AT2 cells were the most proportionally dominant cell type; and third, the non-respiratory viruses had extremely limited capability to target the AT2 cells. These findings indicate the essential role of AT2 cells in viruses transmitted through the respiratory route.

Here, we discussed the probable reasons why respiratory viruses preferentially targeted AT2 cells. First, AT2 cells are located on the gas exchange surface of the alveolar epithelium, which is the critical interface exposed to airborne viruses [57]. Second, as highly metabolic secretory cells, AT2 cells have abundant cytoplasm and an extensive endoplasmic reticulum and Golgi apparatus, which provide important machinery for viral replication; additionally, SARS-CoV-2 leverages the secretory pathway to transport viral particles to the plasma membrane and then is secreted from the cell [58]. Thus, we assume that the physiological location and cell traits might jointly contribute to the preferential role of AT2 cells for respiratory viruses.

SARS-CoV-2 is considered to originate from bats, and its zoonotic spillover to the human population caused the COVID-19 outbreak [59]. In fact, the majority of human viruses are from zoonotic. In the process of zoonotic spillover, the virus population encounters strong natural selection [60], and only limited viruses accomplish interspecies transmission. For overcoming the species barrier and accomplish interspecies transmission, there are two essential factors: (i) the binding of viral entry proteins to the compatible host cell receptors mediates successful viral entry into host cells; (ii) the infected host cells support viral replication and the release of viral particles. Thus, it is well-acknowledged that the binding of viral entry proteins to the compatible host cell receptors is under strong natural selection pressure. Moreover, certain cell types, such as AT2 cells, appear to be more favorable for sustaining viral replication [5, 48, 54]. Consequently, by analyzing the cell-type-specific expression of host cell receptors, certain virus-permissive cell types appeared more often than others, which might reveal the footprint of natural selection that shapes the virus population.

Taken together, the results of this study revealed demographic heterogeneity in the key features of SARS-CoV-2 tropism. This finding indicates that the current definition of vulnerable COVID-19 populations, including sick, male, and older populations, might reflect distinct subphenotypes of SARS-CoV-2 tropism. We believe this finding is essential for improving our understanding of the pathogenic mechanisms of COVID-19. Moreover, we demonstrated that ACE2 expression, the central variable of SARS-CoV-2 tropism, has a causal effect on COVID-19 susceptibility and severity, suggesting the clinical implications of ACE2 in COVID-19 treatment.

To the best of our knowledge, this is the first study revealing the power of comparative tropism analysis for mapping the host cell preferences across multiple viruses. Based on this analysis, we discovered remarkably overlapping and enriched tropism in AT2 cells across different respiratory viruses. These findings have important implications for sociovirology [61], implying conflict, cooperation, and communication among respiratory viruses, suggesting the possibility of coinfection with SARS-CoV-2 and other respiratory viruses. Moreover, with increasing insight into emerging viruses, comparative tropism analysis will help in the understanding of zoonotic spillover events from a novel perspective.
